# Music for Monkeys: Building Methods to Design with White-Faced Sakis for Animal-Driven Audio Enrichment Devices

**DOI:** 10.3390/ani10101768

**Published:** 2020-09-30

**Authors:** Roosa Piitulainen, Ilyena Hirskyj-Douglas

**Affiliations:** Department of Computer Science, Aalto University, 02150 Espoo, Finland; ilyena.hirskyj-douglas@aalto.fi

**Keywords:** white-faced saki, animal–computer interaction, animal technology, audio enrichment

## Abstract

**Simple Summary:**

Animals living in captivity can benefit from new forms of technological enrichment, with auditory enrichment currently being underutilized. Here, we investigate how to provide zoo-housed white-faced saki monkeys with auditory enrichment in an animal-centred manner. To study this, we prototyped and developed an interactive system that the sakis could trigger to play audio and that tracked their interactions with the device. Importantly, we incorporated this system into the regular living environment of the sakis and developed the interaction in a way that gave them control over activating the sounds. Based on the results, we conclude that audio is a promising way to provide enrichment for small primates like sakis. Utilising our device, we demonstrate that the sakis triggered the traffic audio more than silence, rain, zen, and electronic music, with no differences between the other conditions. However, we highlight problems in using this behaviour to infer the sakis preference or how they like the system, with further research needed towards sounds for audio enrichment. Our method reveals the value of collecting early real-world data and prototyping when designing interactive technologies for zoo-housed animals. In this experiment, we found that animal-centred methods can help create technologies better suited to their purpose and ultimately towards the end-goal of improving animal welfare.

**Abstract:**

Computer systems for primates to listen to audio have been researched for a long time. However, there is a lack of investigations into what kind of sounds primates would prefer to listen to, how to quantify their preference, and how audio systems and methods can be designed in an animal-focused manner. One pressing question is, if given the choice to control an audio system, would or could primates use such a system. In this study, we design an audio enrichment prototype and method for white-faced sakis that allows them to listen to different sounds in their regular zoo habitat while automatically logging their interactions. Focusing on animal-centred design, this prototype was built from low fidelity testing of different forms within the sakis’ enclosure and gathering requirements from those who care for and view the animal. This process of designing in a participatory manner with the sakis resulted in an interactive system that was shown to be viable, non-invasive, highly interactive, and easy to use in a zoo habitat. Recordings of the sakis’ interactions demonstrated that the sakis triggered traffic audio more than silence, rain sounds, zen, and electronic music. The data and method also highlight the benefit of a longitudinal study within the animals’ own environment to mitigate against the novelty effect and the day-to-day varying rhythm of the animals and the zoo environment. This study builds on animal-centred methods and design paradigms to allow the monitoring of the animals’ behaviours in zoo environments, demonstrating that useful data can be yielded from primate-controlled devices. For the Animal-Computer Interaction community, this is the first audio enrichment system used in zoo contexts within the animals own environment over a long period of time that gives the primate control over their interactions and records this automatically.

## 1. Introduction

Non-human animals (hereafter animals) have become users of various technologies with the Animal-Computer Interaction (ACI) community exploring the user experience and the interaction that animals have with these systems [1]. These technologies include screen devices [2,3,4], touchscreens [3,5], tangible objects [6,7,8,9,10,11,12,13], olfactory interfaces [14], animal tracking systems [15], wearables [16], and animal-controlled robots [17].

With this growth in research, animal–computer interfaces used in zoo contexts have also increased; predominantly for enrichment purposes [10,11,12,18,19,20,21] and to allow animals to control their activities and environment [22,23]. Here, we define (environmental) enrichment as interventions that enhance the animals’ habitat in ways that support species-appropriate behaviours, introduce stimulating change and complexity in and better control over the environment, or provide cognitive challenges [24]. Enrichment systems in zoos include tablets for apes, designed to provide auditory and visual enrichment as well as cognitive challenges [25,26,27,28]; tracking tangible objects within the enclosure to allow orangutans to generate sounds [19]; digital installations with a projected, motion tracking based interface for orangutans [18,21,29]; a tangible feeding puzzle for gorillas [11]; an interactive cognitive and acoustic enrichment toy for elephants that lets them use different tactile buttons with their trunk to play audio [8,9,10]; a mechanical rhino puzzle feeding ball with a Bluetooth remote controllable via an app [30]; and a remote-controlled rover to evoke hunting behaviour in lions [31].

While there has been an increasing number of computerised systems developed to provide enrichment for zoo animals, there remains some debate on the appropriateness of technology usage in zoo habitats for enrichment purposes, especially when it comes to how naturalistic these solutions are [32]. This issue is closely related to visitor immersion and perceptions of non-naturalistic enrichment in naturalistic habitats [32]. A recent study, for instance, found that when rating pictures of exhibits, people gave more positive ratings when the included enrichment item was naturalistic [33]. Opposing this, positive visitor attitudes toward animals using technology at the zoo have also been recorded [27], especially when technology can be part of fostering engagement and empathy for the animals [18]. Thus, there is a tension when building these computerised enrichment systems for zoos to make them fit within the naturalistic environments of the animals.

Drawing back to systems used in zoos, one instance for technological enrichment are audio systems. The use of audio stimulus has long been proposed as a potential way of diversifying the environment of and providing sensory stimulation for captive animals [34]. However, only a few studies exist within zoo contexts for interactive audio systems [10,12,19,35]. However, the impact and potential benefits of audio enrichment for primates have long been studied in animal science with variable and contradictory results [34]. Ogden et al. [36] discovered rain forest sounds cause an increase in agitated behaviours in gorillas. Robbins et al. [37], on the other hand, found rain forest sounds reduced negative behaviours, also finding that classical and rock music increased negative behaviours with gorillas. Mingle et al. [38] found that chimpanzees preferred African and Indian music over silence. However, for orangutans, Ritvo et al. [39] uncovered that they preferred silence over music and white noise while not discriminating between music and scrambled music, implying no preference. Likewise, monkeys such as tamarins and marmosets have been found to prefer silence and slower tempos over fast ones [40]. Similarly, with gibbons, the presence of music had either no effect on their behaviour or, for some individuals, increased stress behaviours [41]. However, these systems are often tested for very short periods (i.e., a few hours), often not in the animals’ everyday habitat leaving the results prone to the novelty effect.

Typically, audio studies with animals have predominantly used either human music or naturalistic sounds. Building from this, Snowdon and Teie [42] played tamarins both human music and music composed specifically for them. This custom-made “music” contained characteristics based on either the tamarins’ warning calls or affiliation vocalisations. Here, they found that this crafted audio affected the tamarins’ behaviour—but only after the stimulus ended. Specifically, affiliation-based music led to more calm behaviours and decreased activity, while threat-based sounds increased arousal and movement. The human music, on the other hand, had only a subtle effect. As this study and those mentioned above demonstrate, especially using species-appropriate, but potentially also other sounds, can have potential uses as enrichment and directly affect the behaviour of primates. However, there needs to be clear investigation into the type of audio used, how the animal can choose to listen to it, and what this preference looks like for animals.

When studying audio enrichment for primates, experiments often do not give the animals choice over whether they want to listen to the audio, playing the sounds instead directly into their enclosure and analysing the result i.e., [36,37,41,42]. While direct comparisons of voluntary versus imposed audio stimulus do not exist, it has been proposed that improving control that zoo animals have over their environment can lead to welfare benefits [24]. However, most studies comparing primates’ audio preferences that allow elements of choice have been within cognitive science, where typically the participating animals are offered a choice between two audio conditions, albeit in novel settings [40,43,44,45]. Still, these systems and methods (aside from [39]) do not allow the animal to consent (through choice) to taking part in the experiment or to control how they want to listen to the audio. This conflicts with the ethical principles of the ACI community to ensure animal welfare and autonomy by having continuous consent of the animal participants [46]. Noting this, Ritvo et al. [39] built a sound interface for orangutans that allowed them to choose between a sound and silence using a stick as a stylus on a touch screen interface. They termed this method as a participant-controlled procedure, with their key finding being that further work in zoos needs to be conducted in this area [39].

One way ACI as a discipline has approached choice and consent is by aiming to design animal-centred systems that provide the animal as a user the choice to participate and have control over their interaction [4]. Here, animal-centred systems are defined as ones that follow principles for animal-centred research and design laid out by Mancini [46]: respecting all participants, ensuring continuous consent, research focus at improving the welfare of the participating animals, and avoiding harmful procedures. However, how animal-centredness can be advanced beyond these ethical guidelines towards technology implementation, and how the autonomy and welfare of the animals can be supported when they are using new technologies as well as during the design process, are complex problems in zoos [21].

Within ACI literature, there have been many implicit perspectives as well as explicit discussions focused on these challenges. The central perspectives revolve around how to determine whether an animal “likes” or “prefers” a system [35], how animal-centred methods can support the autonomy of the animals through facilitating animals controlling systems [4] and how animals can be included in participatory and co-design approaches [21,46,47]. In practice, prototyping and designing together with the animal users have been explored and found to be an invaluable tool when developing new animal technologies [7,48,49]. These participatory methods have placed the animals in the roles of co-designers in the design process [21,47], evaluators of systems or interaction methods as a form of participation [7,19,48], as well as device users to elicit their interaction requirements and needs [8,9,10,11,18,50].

However, as Paci et al. [50] note, some animals can be stressed by direct participation, so a balance of these different perspectives is needed to find a suitable method for a given context, especially in complex situations such as zoos. Thus, designing technology with and for animals in zoos is a sensitive process. To mitigate this, often the human in the situation (such as the zookeeper, animal behaviourist, and system designer) makes the key early design decisions, with the animal being positioned in an end-tester role, only informing upon their requirements and needs once the system is deployed in the later iterations.

A further challenge to including animals within the design is that when people design computers for animals, approaches and methods can not be directly taken from typical Human–Computer Interaction (HCI) methods created for humans. As Lawson et al. [51] note, animals’ inability to talk with humans to communicate their perspective or reflect and speculate on things renders participatory or co-design approaches utilised in HCI, and, recently ACI, inherently imbalanced in terms of power in favour of the human participants and designers. Along the same lines, Webber et al. [21] acknowledge that animals can hardly be included in design activities requiring creativity, conceptual language, and symbolic representation due to the lack of shared understanding of these concepts. However, they suggest that the animals can be participating in the design as co-designers as *experts of their own experience*, in addition to consulting humans as *proxies* on the animals’ needs as described above [21]. However, this process still involves the animal only later in the design process, even as a co-designer, limiting their ability to contribute meaningfully to the requirements of the technology. Recognising this limitation, Webber et al. [21] point out that, when developing technologies for animals, instead of testing a complete, finalised prototype, it could be beneficial to test out deconstructed features of the system separately to identify specific areas of improvement. Based on these accounts, it can be surmised that including some form of direct participation from the animals can be beneficial while keeping in mind our limited perspective as humans when interpreting the animals’ reactions and behaviours.

However, there remains a grand challenge in zoo technologies for robust methods to design computer systems with zoo animals [21], especially around system aesthetics and quantifying the user experience of an animal [20]. One indicator of this challenge is that technologies used in zoos by the animals have encountered problems in adoption and usage. Typical issues include animals breaking or abandoning systems [18,19,22], which can be a result of missing critical requirements. We propose that a way to meet the key challenge for technologies in zoo contexts could be to further explore centring and iterative processes with animals, seeking to directly involve them early into the design. Especially for the audio context, there is a gap in animal-centred methods for discerning animals’ preference for sound, as well as quantifying the animals’ complex patterns of behaviours to improve user experience and interaction factors for zoo housed animals using computerised devices.

In the case of audio systems, it is common for the animal not to have the opportunity to consent or to dictate their audio preferences. Furthermore, with current zoo-technology systems in general, methods and the resulting systems are generally not designed with animals from the onset, despite the animal-centred focus and steps towards inclusive design. Typically, prototyping is only conducted in the later iterations affecting only the minor details of the system, such as puzzle difficulty [11] or the digital content of the system [29], or it is not clear how insights gained through prototyping directly inform the final prototype or it has not yet been done [19,21,28]. Noticing this, French and colleagues [8,9,10,20] have begun early prototyping of musical devices with elephants to let them inform upon the materials and aesthetics of the system design. From this, they have found that early prototyping with different aesthetics and forms leads to an improved understanding of how animals experience the world, and allows grounding design decisions [20]. This provides a valuable starting point for further zoo-technology work on how to support the early design of systems used by animals.

Addressing the abovementioned gap and key challenges, in this paper, we present a study conducted over several months describing the building and design of an animal-centred audio device for and with the white-faced saki monkey (hereon referred to as saki). We begin by conducting questionnaires and informal interviews to elicit requirements from the keepers and visitors at the zoo. From these gathered requirements, we then prototyped two different structures within the sakis’ enclosure to inform the system aesthetics, form and interaction method from their experiences and interactions. Building from the sakis’ usage and behaviours, we then developed an audio system which uses body tracking to allow the sakis to control the playing of audio autonomously, while simultaneously allowing researchers to investigate and log their behaviours over a period of time. Employing the system, we investigate the audio preferences of the sakis over a longitudinal study from their triggering of the music system.

Through this study, we aim to deepen the methodological understanding of how zoo animals can be more included within the design process throughout the design and prototyping phases, and the ramifications of this inclusion. Here, it was found that the developed audio system is shown to be useful long-term to track system activation behaviour and to explore what happens when the sakis are given control of an audio device. We reflect upon both the method itself, the design process and what was learned from the sakis’ interactions with the system. Our two research questions related to these objectives:

**RQ1:** How to design audio enrichment devices with an animal-centred focus with the white-faced saki?

**RQ2:** If given a choice, do the white-faced sakis as a troop have different preferences for various sounds or prefer silence?

The impetus for primate-controlled systems is to explore how animals might use future interactive audio-based devices, especially in unsupervised autonomous ways. Systems and the surrounding method, like the one presented here, could provide audio enrichment for zoo housed animals and provide them with a way to augment their enclosure and explore sounds freely. With zoos having limited resources and zoo animals limited freedoms, this paper explores positioning the animal as an active user without the need for human intervention. The presented method provides a way towards user-centred design by including the animal throughout the iterative process, creating more usable and accessible animal-technology systems.

## 2. Materials and Methods

This section is divided to two parts: the first describes the requirements and design of the audio system structure, while the second part focuses on the interactivity and methods used to compare the audio preferences of the sakis. In summary, from our initial requirements, we started by designing two non-technical prototypes to see the frequency and type of interactions the sakis would have with different forms, aesthetics and shapes. Informed by the sakis’ interactions, we then picked the form that was used most frequently and supported complex and diverse behaviours. From this shape and materials, we then built the mechanism that the sakis can use to interact with the audio system based on their ordinary interactions with the non-technical prototype. To test this system, we then employed and examined the sakis’ interactions (both frequency and length) with different audio outputs. This whole process is expanded upon below.

The sakis participating in this study were maintained at Korkeasaari Zoo, and all of the experimental procedures were ethically approved by the zoo themselves. The sakis were one of two primate species that the zoo offered as candidates for this study, and were ultimately selected due to their small size which made it feasible to create a prototype that could safely be added within their exhibit over a longer period of time. Moreover, sakis have not previously been investigated with audio enrichment, helping to bridge findings for primates and audio.

The participating troop of sakis within this paper consisted of seven individuals (M = 9.7 years): three castrated males (aged 21, 4, 3) and four females (aged 22, 11, 4, 3). The ambient soundscape of their habitat included vocalisations of other animals living in the Amazonian house section of the zoo (where they lived), mainly bird calls and sounds of other small monkeys. The sakis’ enclosure consisted of two adjacent spaces that are on display for visitors, each approximately 9 × 6 meters. The space in which the prototype was placed consisted of a large space with a central tree, various hanging logs and branches, plants and other enrichment devices. The sakis can freely move within and between these spaces. In the summer months, they also had access to an outdoor enclosure but were housed indoors during this study (winter period). The sakis had no previous experience with music or interactive technology devices.

When it comes to assessing preference of animal users, it is a complicated question with a variety of approaches. Since it is impossible to know what the animals like and what liking means to them, as researchers we have to work around this and make inferences based on some behavioural aspects and what is known about the animals in general. Ritvo and Allison [35] describe three approaches to assessing preference for primates in this way: behavioural observation during stimulus exposure, the least-aversive or most-desired choice paradigm of forcing a choice between two options, and participant-controlled procedures where the participating animal can choose the length of exposure to the stimulus. Throughout this paper, we utilise the participant-controlled method and let the sakis freely interact with prototypes as they wish, using the amount of interaction as an indication of preference building from prior work [4]. However, what this preference means, and its applicability towards liking will be unpacked within the discussion. Thus, the term preference here is used to differentiate which audios the sakis triggered or which forms they used more.

### 2.1. Requirements and Forming the Prototype

To form the initial system in an animal-centred manner, we firstly gathered and formed initial requirements. We started this by observing the sakis and informally interviewing the keepers working directly with the sakis upon the challenges and opportunities for technology enrichment devices to get a more comprehensive understanding of the animals’ needs and behaviours as well as the context of use. Additionally, a requirement gathering questionnaire was handed out to zookeepers and those working with the sakis at the zoo. The same questionnaire was also distributed to visitors by the saki habitat to include their perspective, as zoo visitors observe the animals and have shown to influence the zoos’ usage and aesthetics of technology systems [27,33]. Thus, while visitors are not experts on animals, their opinion does directly affect aspects of the technology used by animals [32].

The questionnaire included questions on how the prototype should function, aesthetics and their views upon the wants and requirements for technology enrichment systems on behalf of the sakis. This included a total of twenty-two questions: five questions about demographics and visitor experience, seven Likert scale questions, four of which had an additional optional open question for elaboration, three selection questions and three open-ended questions. Link to the complete questionnaire can be found in Appendix A.

The questionnaire had 56 responses; 52 from visitors and 4 from zookeepers who looked after the sakis. The responses were grouped and coded into requirements. Themes from the scaled questions were included if the median response was non-neutral, and selection questions if one option was clearly preferred over the others. Open questions were analysed using thematic analysis, and frequently appearing themes (mentioned by more than five people for visitors or a single keeper) that were not presented in the other questions were also put forward as a requirement. Keeper and visitor requirements were first formed separately from each other as the keepers are experts, and then combined to form common requirements (mentioned by both the keepers and visitors). These final requirements are listed in Table 1.

Since the starting point of the project was to create some type of interactive enrichment for the sakis, a decision about the specific type of enrichment was finalised after this initial requirements formation. This was done by informally speaking with the zoo staff (research coordinator, the curator of the tropical house, the main keeper of the sakis, and a biology researcher who had been working with the sakis). Here, they stated that the sakis did not have any particular previously unmet needs that the technology should address. With the zoo experts, ultimately the audio concept was selected because the sakis were already receiving food-focused enrichment. Thus, using sound would provide them with a different type of enrichment that they had not previously experienced, and an enrichment method which is underutilized in zoo contexts. Furthermore, not including food as a reward or motivation also enabled a system that is freely available for the sakis, since access to food cannot be unlimited to keep the animals in a healthy weight.

The audio enrichment as a concept fulfils the enrichment requirement (CR1). Health support (CR2) in our questionnaire was considered as a general concept of supporting the health monitoring of the animals, and in the open questions health support was often proposed without the respondent specifying any particular dimension or specific application. Regardless, the audio system does not directly address this requirement. One of the central challenges for our concept was to ensure the system has no unintended negative consequences (CR3), e.g., it needed to be safe for the animals to use. Additionally, as a negative consequence specific to audio enrichment, we considered how the other animals in the troop might involuntarily hear the audio stimulus triggered by another animal and react negatively towards the interacting sakis’ choices. Thus, the design of the prototype was required to be such that the animal listeners (sakis triggering the audio interaction) should be the only members able to hear the audio. Regarding the interaction mechanism itself, we chose to have no training involved to fulfil CR4 and reduce frustration (KR2). Here, the keepers were concerned that a system that would be hard to understand or otherwise frustrating for the sakis to use might affect them negatively. The system being too easy (KR3) on the other hand related to puzzle type enrichment systems, where it was seen that any such system should be appropriately challenging. The system also had to allow for monitoring the sakis’ use to ensure its safety (KR5) and not to be addictive (KR4) to prevent any compulsive habits with negative consequences. With audio, this was not a known issue so we assumed addiction to be a minimal risk factor. Additionally, the system structure had to be able to withstand the sakis’ biting and jumping behaviours (KR1). The keepers elaborated on this further during our interviews, mentioning that the materials and structures had to be safe and non-slippery for the sakis. Speculating more upon the form during the interviews, the keepers felt the designed form should not be too confined as they worried about the sakis feeling trapped. In addition to these requirements, building from prior work and methods [39,52], we required the system to enable the sakis to have consent and control over the interactions.

**Figure 1:**
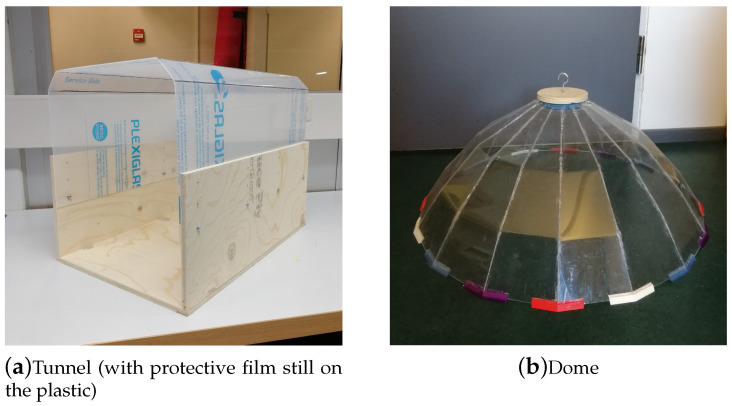
The initial non-functional prototypes.

Based on these requirements, we formed two non-functional prototypes to investigate the sakis’ reaction to different shapes and materials (Figure 1). Testing these lower-level prototypes with the sakis was done to ensure further animal-centred design decisions. In this way, we aimed to iteratively design with and for the sakis by building the enrichment system from their interactions.

The two prototypes formed were *tunnel* and *dome* shapes (Figure 1). The first prototype, coined *tunnel*, consisted of a plywood floor and partial sides and a transparent 2 mm acrylic (plexiglass) rounded hood to form a tunnel-like shape to prevent the sakis from feeling trapped (Figure 1a). The *tunnel* form and materials fit with the requirement of isolating the sound with good acoustic properties without having a fully enclosed space. The materials used (wood and plexiglass) were familiar to the sakis and chosen to be non-slippery on the floor, fulfilling the keeper requirement.

The second prototype, coined *dome* (Figure 1b), was a 60 cm dish form that was designed to be hung in the enclosure. Unlike the *tunnel*, the *dome* did not require the sakis to enter an enclosed space, addressing the keepers’ concerns. The *dome* form was also made from 2 mm plexiglass formed into a parabolic shape that could directionally reflect sound (1100 Hz and above) to only the specific area below.

**Figure 2:**
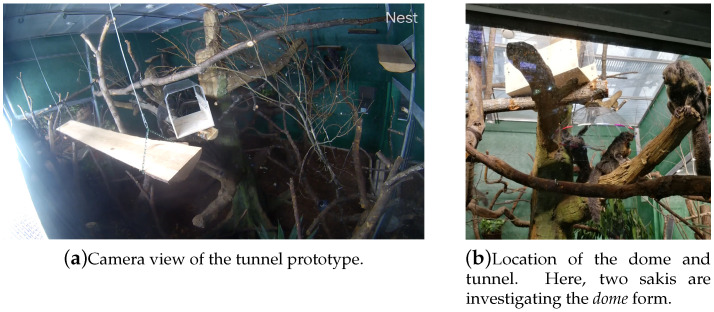
The prototypes in the enclosure.

To test both these forms, the prototypes were introduced to the sakis’ regular living enclosure and observed over a period of sixteen days. This period was chosen to be long enough to become part of the sakis’ everyday environment. For securing the new *tunnel* enrichment system, a new branch was added approximately 3 m above the ground. The *dome* form was hung from the same branch, with a branch below for the sakis to sit on being approximately 2 m high. Both these prototypes were put in the middle of the larger of the visitor-facing spaces, where the sakis were able to approach them from several directions and interact with them easily and freely (Figure 2).

Observation of the sakis’ prototype usage was done systematically through video recordings via a camera within their enclosure (e.g., Figure 2a) as well as in person through observing their enclosure. The camera interface automatically tracked and highlighted the sakis’ movements surrounding the prototypes. We then verified these initial logs through watching the video recordings and transcribing the sakis’ interaction timings, frequencies, and the number of animals participating in the interaction. Each interaction was then encoded with codes describing the interaction (Table 2). The process of coding behaviours from the video material was developed through thematic analysis by the two authors, allowing the data to be grouped across the behaviours detected. The final codes consisted of physical behaviours (*grab, bite, touch*), the animal’s position around (*over, on top* and *next to*) and within the device (*through, edge* and *sit middle*), other (such as behaviour around the device and food eaten in the system context) and social behaviours (*groom*, *shoo* and *multiple individuals*). Here, the *shoo* label refers to a saki approaching the device causing another to leave. Each interaction instance was labelled with all fitting codes for the whole duration resulting in total percentages above 100%.

A total of 677 interactions were recorded with the *tunnel* prototype (average 42 per day) and 54 interactions with the dome form (average 3.4 per day) (Table 2). For the *tunnel* prototype, the average length of a single interaction was 20 s (median 4 s), while the longest interaction was 16 min and 38 s. The average length of the interaction with the *dome* form, on the other hand, was 13 seconds (median 8 s) with the observed maximum duration being 88 s.

On the first day with the prototypes, the sakis interacted more frequently with both prototypes in comparison to the whole testing phase. This indicates high novelty in initial exploratory behaviours which reduced over the experiment period. With the *tunnel* form, the sakis initially investigated the artefact through biting the screws and the sides of the *tunnel* with *grab* behaviours being present in almost half of their initial interactions (40%). For the *dome* form, the sakis would reach up and touch the artefact (84% of all *touch* interactions happened during these initial two days).

**Figure 3:**
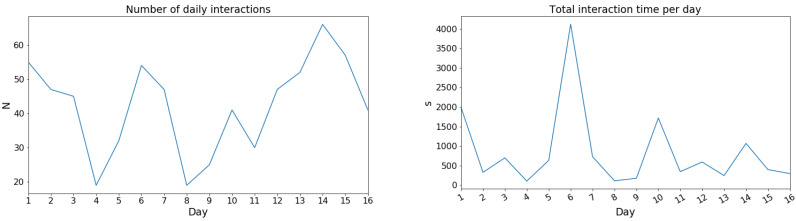
Daily variations in the tunnel prototype usage.

After this initial curiosity phase, there were clearer differences in the sakis’ interactive behaviours towards the two prototypes (see Table 2). The *dome* form was largely ignored after two days, with the only behaviours in this period being the sakis sitting under the prototype and rarely touching the artefact (Table 2). For the *tunnel* form, the interactions were variable both in frequency and length. The high interactive periods often consisted of a singular long interaction (up to 10–15 min) when a saki would sit or sleep inside the form (see Figure 3). The grabbing and biting behaviours as forms of exploratory interaction continued, though these became less frequent.

There was also minimal social use of the *tunnel* form, where two or more sakis were using the prototype at the same time or together including behaviour such as *grooming* and *shooing* in moments of co-existing (Table 2). Other behaviours included bringing food and eating on or in the tunnel (*food* in Table 2), or looking closely through the transparent part while sitting inside (*look through* in Table 2).

In summary, the sakis’ investigation was physical with new objects in their habitat and there was a noticeable novelty effect. The *dome* form was ignored by the sakis after this initial investigation, while the *tunnel* form was adopted as a daily location supporting a higher level of interactivity with the sakis exhibiting multiple behaviours, including social behaviours with and around the prototype. Using the amount of interaction as an indicator of preference following the participant-controlled procedure [35], we thus selected the *tunnel* form for further development. A potential confounding factor for this preference for the tunnel was the location. However, using the dome would not have been practically viable in the same location as the tunnel, since hanging it from the ceiling was not possible. Furthermore, the tunnel supported versatile behaviours and interactions, as well as social usage (which was noted to be desirable by one of the sakis’ keepers) during the test period. In this manner, we built from the sakis’ interactions themselves as a way of involving them in the design process, taking guidance from the people involved in the sakis’ lives. The remainder of this paper will focus on building the technical form and the testing of this device.

### 2.2. Interaction Method

Building from the form, the technological version of our enrichment device had to provide audio stimulus triggered by the sakis; in short, whenever a saki was detected within the tunnel, audio would play and the interaction time and duration be logged. When designing the interaction mechanism, the sakis behaviour and physiology, as well as previous literature, were considered. Observation and conversations with the keepers yielded valuable information on how this specific troop of sakis interact with their environment. For instance, we were informed that, when target-training the sakis, some of them like to touch the targets with their hands, while some prefer their whole face or their nose. They also do not use tools or very sophisticated hand-interactions but tend to bite things or grab objects with their whole hand.

These insights helped us limit our options to the most viable ones. For example touch screens, which are widely used also for animal interfaces with e.g., dogs [3] and orangutans [28], might not be the optimal solution since the sakis’ preferred interactions are not as precise as touching things with fingers. Also tool based interfaces, such as used before with gorillas [11] were ruled out. Other methods used with animals to trigger events include using a proximity trigger ([4] with dogs), as well as tangible interfaces such as a rope pulley system ([6] with dogs) and tactile interfaces ([20] with elephants). We did not use [6] and [3] methods as these required extensive training to enable interaction, going against our requirements (CR2, see Table 1). Furthermore, training has previously been found to be a confounding factor with audio interfaces [39]. Thus, we chose the proximity-based approach similar to [4] as this provides the animals with a simple and straightforward association between the trigger (distance) and the audio stimulus. This additionally enabled using the time spent inside the *tunnel* as a measure to indicate triggering of the devices as preference as noted prior [4]. This interaction method also allowed the sakis to stop the interaction at any time through leaving or avoid the interaction altogether, providing them with a form of consent to use the system as defined by [46].

### 2.3. Forming the Technical Prototype

**Figure 4:**
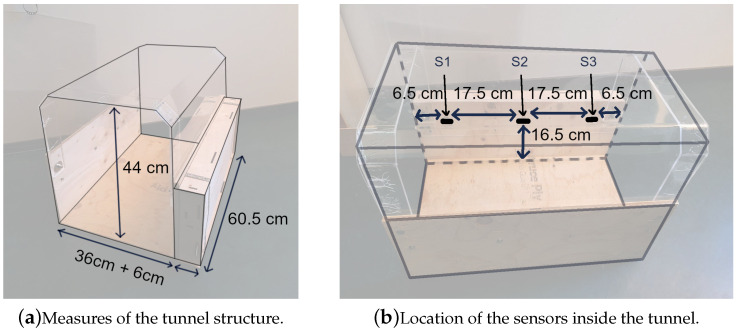
Measures of the tunnel prototype.

**Figure 5:**
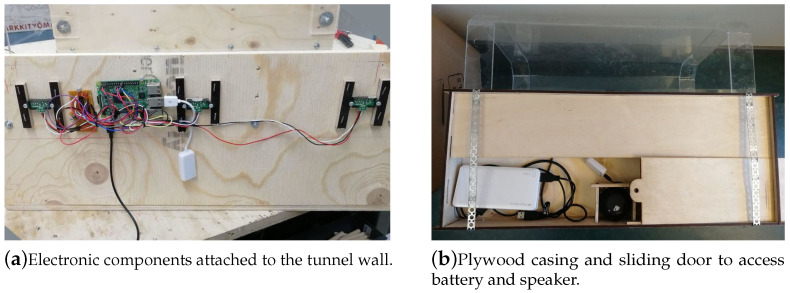
Electronics attached to the tunnel.

For the hardware, a Raspberry Pi 3 with infrared (IR) distance sensors (SHARP GP2Y0A41SKOF 4–30 cm) was used for proximity tracking. IR was chosen because it works with the sakis’ fur and can detect the presence of both stationary and moving animals. Passive infrared (PIR) and ultrasound sensors were considered as an alternative, but PIR detects movement rather than distance (presence) [53] and ultrasound does not work ideally with furry animals [54]. Three IR sensors were placed along the tunnel to create an interactive zone so that any animal going inside or through would be detected (Figure 4b) and the audio triggered every time it happened. Each sensor was set a threshold customised to the location with which the animal was reliably detected when inside the tunnel to trigger the audio. The system described above was mounted outside the original tunnel form inside a wooden casing, and holes were made for the IR sensors and speakers (Figure 5). The speaker used was an XMI X-Mini II mini, with the speaker set at 100% volume and the volume output level set to 50% from the Pi. This allowed the audio to be audible by the sakis but contain the audio within the structure. As the system resided inside the sakis’ enclosure, it was powered by a rechargeable battery pack which was accessible via a sliding door. Specific measures of the finished tunnel prototype can be seen in Figure 4.

For recording the interactions, the interaction length and timing were logged in real-time locally and to an online worksheet using a Python script to allow remote inspection and automatic recording. Additionally, to allow remote monitoring of the system, the device updated its status (online/offline) every five minutes to our online workspace. Additionally, the Pi could be accessed and updated remotely, allowing the sound to be changed and the systems’ software modified without entering the enclosure.

There is no definitive data upon sakis’ hearing capabilities, but Henline [55] found their vocal range to span about 900 to 7000 Hz in fundamental frequencies, with some formant frequencies reaching up to 17,000 Hz. Out of these, the higher frequency whistles were typically related to solo activities, such as feeding or object manipulation, when around other individuals. Alarm calls, on the other hand, spanned the lower end of the frequency spectrum. When selecting the sounds, we avoided sounds that would explicitly contain sounds of their predators. Furthermore, we discussed what sounds might trigger a negative reaction with the zoo staff. Based on this discussion, it was determined that, as long as the audio used did not contain any extreme sudden noises, preemptively avoiding potential negative sounds was not necessary since the sakis had the freedom to move away from and completely avoid the system.

**Figure A1:**
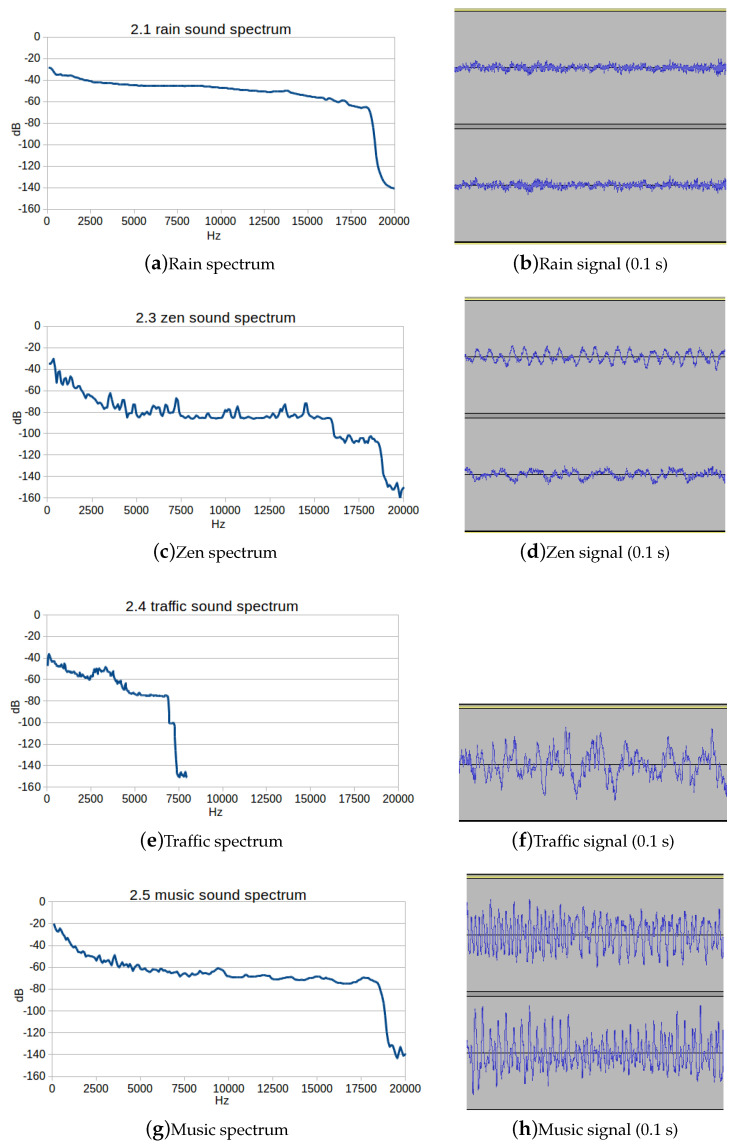
The spectrums of the test sounds.

Ultimately, the four sounds used for the system were *rain*, *zen*, *traffic*, and *electronic music* in this order. Since not much is known of the audio preferences of primates, we selected sounds with diverse overall spectrum and temporal variability (see Appendix B, Figure A1 for more detail upon the spectrums). We additionally included sounds with constant background noise to allow for consistent audio stimulus in response to the triggering to make the interaction clear. All the sounds were played in a loop so that every time the animal activated the audio it resumed playing from where it had left off, and looped back to the beginning when the track ended.

The *rain* (01:36) sound was selected initially as it was unknown how the animals would react to audio in general. Rain was a familiar sound to the sakis, as they had access to an outdoor enclosure during summer months. The selected rain track was generic, quite uniform background rain noise with occasional more clear splatters of individual droplets, with minimal temporal variation and the spectrum being close to pink noise. The second sound tested, *zen* (04:24), was an electronic ambient music with occasional short wind chime-like and string instrument jingles and melodies. The overall spectrum of this track was more varied than for the rain sound, but the temporal variation was still mild with no sudden loud sounds. The *traffic* (04:39) audio was a recording of a busy motor traffic in India, including vehicle honking and engine sounds with a general background hum of traffic. The overall spectrum of this track was the most diverse with distinct temporal variations from the honking but had uniform background noise. However, the frequencies of the track cut off around 8000 Hz, thus it did not contain any higher frequencies and is not representative of normal traffic frequencies. The fourth sound tested was the *electronic music* (02:14) condition of an electronic music track with a clear melody, rhythmic bass beat and high tempo. Since previous studies have indicated that primates do not prefer human music [37,38], we included a more traditional choice to create comparable results across studies and methods. The spectrum of this track was more varied than zen sound with steeper low than the rain sound. Furthermore, the temporal variation was the most prominent but with fewer spikes and more organised than the *traffic* sound.

### 2.4. Study Method

For the method of testing the sakis’ behaviours in reaction to different sounds, we used a method of a control period of silence (no audio) to form a baseline followed by periods of audio stimulus [4]. The audio stimuli were provided in seven-day iterations to gather the weekly routine of the sakis’ enclosure, playing each sound in sequence without time off in between. However, the system suffered from occasional downtime (as elaborated in the limitations section) and thus to record seven days of data for each of the conditions, some of the iterations spanned more than seven days to account for the down time. Consequently, in total the system was used over a seven-week period with the results normalised by the total number of hours.

After forming the prototype, the technological system was placed in a lower position in the enclosure to allow for keepers to maintain the system (as it required daily battery changes) and with the same troop as described above. This was not ideal but done out of necessity since the original placement proved to be too unpractical to maintain. Comparing the silent condition in these two locations, we found no significant difference in the total mean interaction time (Wilcoxon signed rank sum for mean, W = 149.0, *p* = 0.506, f = 0.500). All the data presented and compared in the results section is collected from this lower location, including the silent condition. Only the initial prototyping (as described above in Section 2.1) was conducted in the initial location. We additionally did not analyse the sakis’ general behaviour for the technical prototype, only their triggering behaviours.

#### Data Analysis

The raw data logged from the sensor readings consisted of a timestamp and Boolean flags for the status of each sensor as well as a flag indicating whether this timestamp marked the beginning of an interaction. From this raw data, interaction times were constructed in the form of start and end timestamps and the duration of the interaction in seconds. The data were cleaned of false-positive sensor readings by dropping events shorter than one second since any true interactions involving the sakis were not shorter than this. The validity of sensor readings was confirmed by selecting random interactions (*n* = 25) and verifying their correctness from the saved video records.

Since it is not obvious and previously unknown in what way the sakis would trigger the audio, three different measures of interaction were used: total interaction time, frequency of interactions, and the length of individual interaction. In this way, we hoped to get a more complete understanding of the usage patterns and interactions the sakis have with the system and how the different sounds might affect that. For instance, the sakis might prefer frequent but short interactions or fewer but longer interactions, and using only one of these three measures would reveal only one aspect of this dynamic.

Wilcoxon signed-rank test was used to compare the interaction times in different conditions and descriptive statistics for indicators of activity. Due to the confounds of the study location (the zoo environment), there was some variation in how long each sound condition was tested; however, for each condition, the system was on during a minimum of seven days. This was primarily due to the system being powered by batteries within the animals’ enclosure. Occasionally, the zookeepers forgot or did not have time to change this battery and the system would power off and have to be restarted manually, leading to some downtime during the test periods. As a result, the descriptive statistics of the number of interactions and the total interaction time are normalised by the testing period (hours). As the device triggered on a saki’s presence, even the short interactions of walking through the tunnel would trigger sounds. Thus, the Wilcoxon signed-rank test was chosen because the data was heavily skewed, most interactions being just one second long (i.e., a saki just walking through the tunnel) and the number of interactions decreasing exponentially as a function of the interaction duration.

Additionally, as the group of animals (sakis) was the same in all the conditions, the samples could not be assumed to be independent. Thus, a Wilcoxon signed-rank test was used to calculate the interaction duration in 30-min intervals to avoid dependence of the pairs in a long enough interval. Additionally, since the test requires pairwise data, the 30-min intervals were averaged over the test days. With this set-up, every condition was compared to each other pairwise with the $H_{0}$ being that the distribution of pairwise differences has a mean of zero. The alternative hypothesis $H_{1}$ was that the mean of differences is positive, i.e., the values are greater in favour of one condition of that particular test. Link to the data and code used for analysis can be found in Appendix A.

## 3. Results

The results are presented below according to our two research aims; the first is evaluating our audio enrichment system and the method used in regard to an animal-centred focus, and the second was to discover audio preferences for sakis vs. silence. As the technical version of the system was in place for several weeks, it was possible to track the sakis’ activations and system usage over time, Figure 3). This tracking exposed initial key findings. First, the device was used by the sakis for various social behaviours and as part of their everyday environment, making the design of the system and our method workable and suitable to track and deliver audio stimulus controlled by the animal themselves. Secondly, there is evidence that the audio used to augment the space changed the way the sakis triggered the device, indicating that they may have different preferences for different sounds.

When the sound was first added to the system, the sakis were slightly spooked by the change and would run from the tunnel when the audio was triggered only to return to investigate. Within some minutes, they were looking for the source of the sound and closely inspecting the wall of the tunnel behind which the speaker was mounted. This initial investigative behaviour, however, largely stopped within an hour, after which the sakis’ behaviours with and around the system went back to what they were before adding the sound.

The data revealed no significant differences between the sakis’ interaction time of the device usage between the *silent*, *rain*, *zen*, and *electronic music* sound conditions (Table 3). However, for the *traffic* condition, there was a statistically significant difference where the audio system was used significantly more compared to any other condition (*p* < 0.01 when compared to *silent*, *rain*, *zen*, and *p* = 0.015 with *electronic music* condition, Table 3). The effect size for *traffic* sound was largest compared to *silent* with 75% of the time intervals having longer average interaction duration when *traffic* sounds were played. Thus, from just our interactivity data, the audio and audio type had an effect on the sakis’ usage of the device. Here, out of the tested sounds, the sakis had the least interactivity with the device with the *electronic music* condition and the most with the *traffic* audio condition when it comes to the number of interactions.

Looking further at the interaction length and amount of interactions, they were very skewed for all the conditions (D’Agostino–Pearson’s test for normality *p* = 0). The median length of a singular interaction with the device, however, was between 3–6 s for all sound and no-sound conditions. However, there was considerable variation in means and standard deviations (Table 4). Thus, the sakis used the *tunnel* prototype in different interaction patterns depending on the audio. Compared to the audio conditions, the *silent* condition where the system played no music had a high mean (23.35 s) and especially standard deviation (107.5 s). Thus, while the device was used the least in terms of frequency, its usage time was higher than for all sound conditions except for *traffic*. The *traffic* condition, on the other hand, had the longest duration of interaction (std = 73.79, M = 28.77). The *rain* and *zen* audio conditions were lowest for interaction duration.

Regarding the number of interactions per hour, this supported the results of the significance test with the number being the highest for the *traffic* sound condition (Table 4). For the other audios (*rain*, *zen*, and *electronic music*), the frequency of interaction were similar and slightly higher than for *silent*. Notably, the total usage per hour was shorter for the other audio conditions than for both the *traffic* sound and *silent* condition (Table 4).

Investigating the long interactions, the *silent* condition had occasional long periods of interactivity, but few of these exceeded 900 s (15 min) (longest for *silence* was 1052 s; Table 4). The longest interactions for sound conditions *rain*, *zen* and *electronic music*, on the other hand, did not go much beyond 300 s (5 min) (rain 116 s, zen 141 s and music 307 s—Table 4). This resulted in the *silent* condition having the least number of interactions per hour (1.84; Table 4), but longer total interaction duration than the aforementioned sound conditions (1052 s; Table 4). The notable exception to the other sounds was the *traffic* sound, which had the highest average number of interactions per hour out of all the conditions, as well as longest individual interaction time. This further indicates that the *traffic* sound was triggered, and thus preferred, to the other sounds followed by *silence*. Regarding the other sounds, these were then not preferred to the *silence* condition.

**Figure 6:**
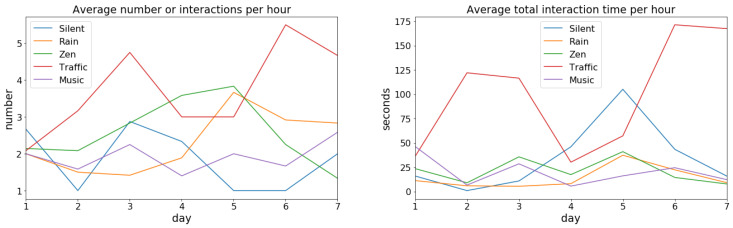
Daily variations in the tunnel prototype usage.

Looking closer at the day-to-day interaction, this varied daily, especially with the highest interactive conditions of *silent* and *traffic* (Figure 6). There were no obvious patterns to this variation between the sound conditions. Furthermore, the days with the longest total interaction times did not always coincide with the highest interactivity days for each condition. This was especially the case for the *silent* condition, where, for example, day five had total interaction time double that of any other days, but the average hourly interaction count for that day was only one. Thus, this indicates that both the interaction frequency and total use time need to be investigated to get a full picture of the interactivity and the patterns of use.

## 4. Discussion

The findings of this study contribute knowledge of methods and how to design systems to study a saki’s interactions with an audio device (RQ1), as well as what audio sakis choose to listen to in their everyday environment in the zoo (RQ2). The following discussion will explore these themes, situating our findings in prior animal computing research. We then present limitations and suggestions for future work.

### 4.1. What Was Learned about the Method and Designing with Animals in Zoo Environments (RQ1)

The method employed to design the audio system and to track and record the sakis’ usage of the device was animal-centred, in that the participating sakis were given autonomy over their participation and were free to choose to not use the system, the system was in use in their everyday environment, and their responses to the prototypes were used to direct the design. Furthermore, the system was effective at reporting and gathering data of the sakis’ interactions. The sakis were consistently using the system throughout the study, indicating that they were comfortable with the device in their everyday enclosure.

The method itself allowed the system to automatically remotely report the animals’ interactions without physical inspection, minimising the need for maintenance from people and thus disturbances for the sakis. As the sakis’ interactions with the system changed over time, the longitudinal approach mitigated against the day-to-day rhythms of the zoo environment providing accurate data. In particular, the sakis’ initial interactions with the system consisted of biting and grabbing behaviours, which the keepers noted were typical exploratory behaviours. After a few days, these reduced and the sakis started to exhibit social and relaxed behaviours, such as sleeping and grooming, within the system. These behaviour patterns would have likely been left undiscovered if the system had been tested only over short periods. Thus, by giving the sakis ample time to explore and get used to the system in weekly iterations without any food incentive, we ensured that the data reliably reflects the long-term use patterns independent from novelty effects, keeper and other human influences, and food biases. This builds on prior zoo technology systems, which often only test for short periods [11,18], potentially obtaining biased results and leaving behaviour patterns undiscovered when animals are not allowed to fully experience devices and systems. Thus, akin to Grillaert and Camenzind’s [56] comments on pet-technology, we advocate and provide evidence that conducting experiments and collecting data over a long period of time in real contexts provides more accurate and normalised data.

As a part of our method, we included the animals early on within the design process to help form the interaction mechanism and investigate the sakis’ interactions with the form of the system separate from the audio stimulus. While French and colleagues [8,9,10,20] have previously undertaken prototyping with animals (an elephant), no prior work in zoo environments has included multiple animals in this manner. Collecting this real-world data of the animals’ behaviours and responses during the design phase, before including technology, allowed us to direct the system development towards this user data, instead of relying solely upon the knowledge of the caretakers of the animals as is often done.

Insights from early prototyping of low fidelity non-interactive versions cannot necessarily be taken as a guarantee of the same behaviours and interactions being exhibited when prototypes are made interactive. After all, it could well be that the animals would rather interact with technology differently than with a purely physical, non-responsive object. However, in our study, this early prototyping proved to be essential, as the expert knowledge provided by the keepers concerning the shape form (dome over tunnel and avoid enclosed spaces) and the sound preferences (avoid traffic) did not ultimately correspond to the animals’ true reactions. By prototyping with the sakis, we allowed their interactions to inform us when forming system requirements and the design of the form, resulting in an animal-centred method. Along the way, we also discovered that the initial structure on its own could also be a valuable addition to their environment, judging by the frequency and diversity of their usage of the prototype. In this manner, our method can provide useful information that can be used to augment an animals’ environment, even if the final technological system proves not to be ideal for use.

Additionally, as our initial prototype forms demonstrate, the form of the device itself heavily influences the sakis interaction style and frequency. Since we tested the structure already without the interactivity, we were able to separate the behaviours in response to the form from the ones in response to the audio; had we directly implemented an interactive system and included the sakis only after these decisions were already made, we would have had the problem of separating the effect of different aspects of the prototype, as Webber at al. [21] reported on their experiences with prototyping. This highlights the importance of making system and design choices motivated by data to move away from human bias. However, we recognise that this does not fully remove the problem of bias, since human interpretations are still necessary to make sense of the data. Here, the advice of Lawson et al. [51] to stay aware of our humanness and lack of understanding of the experiences of other species is relevant to avoid anthropomorphised inferences from the data.

Despite this, even though the participation of animals in design does not look the same as with humans, is inherently more limited and brings its own challenges with it, we find there is value even in this limited form of participation. If we had relied solely on the expertise of the caretakers of the sakis and directly implemented a system using the dome structure, ultimately, we would have likely ended up with a system that was used less by the sakis as shown through their interaction with the non-technical forms. In this way, by including the sakis in our method of participation, we were able to explore a wider design space and make decisions based on insights from observing the sakis. In our study, we view this as participatory design, as we approach the animals directly in addition to the indirect approach of involving humans as proxies, and let their interactions guide the design already early on. The initial requirements of the sakis are indirectly inferred through the expertise of the keepers and our own experience as interaction designers. However, valuing the sakis as stakeholders, we then create opportunities for the sakis to directly engage with early prototypes before all the key design decisions are made (such as interaction type, form). This allows us to see how the sakis approach these structures and incorporate information from their interactions and behaviours to the next iterations of the design process. Thus, we argue that even limited forms of participation that may not be the same as methods or processes used with humans, can be useful to inform critical early design decisions, when implemented in a way that animal welfare is ensured and the prototypes are created based on the best possible knowledge on the animals’ needs and characteristics. We suggest that in this way we can take steps towards more animal-centred design practices without making the participation of the animals an empty facade as Lawson et al. [51] have commented.

Reflecting further, another way to empower the animals and ensure a more animal-centred approach is to provide them autonomy and choice over interacting with the system [2], adhering to ACI principles [4,52]. This choice, or lack of, has affective, motivational and physiological effects on animals, especially with monkeys [57]. Building upon Ritvo and MacDonald’s [39,57] work, our method allowed the sakis to use the system if and when they wanted to, providing the animals with autonomy and giving us an indication of continuous consent. By combining this choice paradigm with proximity detection as a form of interaction method, we are building upon prior methods by Hirskyj-Douglas and Read [2] for pets towards zoo housed animals. The presented method allows the animals to have this control within their normal environment, with minimal human intervention or inclusion.

### 4.2. What Was Learned about Audio Preferences for White-Faced Sakis (RQ2)

One of the key contributions to come from this study was what audio the sakis choose to listen to and how their interactions with the system looked in terms of frequency, length and day-to-day variance. Previously, audio studies with primates have mostly used human music or white noise [38,40,41,45] having shown indifference to audio or negative reactions even to natural sounds [36]. Our findings for the zen, electronic music and rain conditions align with this. The single clear deviation, however, was the *traffic* noise, during which the system was used more than any other sound condition (including silence) both in terms of duration and frequency of interaction. This result serves as an example that, when looking at animals and their preferences for audio, it is important to include various different sounds and not just ones that are hypothesised as being pleasant for people or animals alike.

As for why the *traffic* audio was triggered more by the sakis, our study design does not allow drawing any definite conclusions beyond this. One potential explanation for this could be that the audio track did not contain any high frequencies above 8000 Hz or that there are some features of the *traffic* sound that resemble the vocalisations of the sakis, which could influence the sakis’ preference as has been found previously with tamarins [42]. While there might be some unknown confounding environmental or other factors that caused this difference for the *traffic* sound, we consider this unlikely. There were no known apparent differences to the other conditions in the sakis’ environment during this period, and the week-long test duration would compensate for any drastic daily variations due to the normal variance of the zoo environment. Furthermore, both the number of interactions and the average interaction times were consistently highest for the *traffic* sound across almost all of the test days (Figure 6), which indicates there should have been a consistent coinciding variable during specifically the *traffic* sound test period. Thus, our results indicate that the sakis prefer and seek to listen to certain sounds, but it does not identify the features of the audio that influenced this preference.

Additionally, while *silence* was preferred in terms of the length of the interactions to the other sounds except for *traffic*, the number of interactions was higher for all sound conditions over the *silence* condition (Table 4). Looking further into the type of interactions the sakis had, the interactions were typically very short (1–4 s; Table 4), which is much less than previous research audio clips played to primates i.e., 10 s [45]; 30 s [39]; 40 min [38]. Thus, while we initially found that sakis prefer shorter interactions, as [2] found prior with dogs, further speculation is needed for primates. However, what this does indicate is that in future audio enrichment studies, the systems need to have this flexibility for short and long interactions. The longer interactions that the sakis had often involved social and resting behaviours such as sleeping and grooming. Thus, there is a difference in the interactivity patterns (in terms of the length of usage and number of interactions), despite no statistically significant differences, for most sound and non-sound parameters. These differences in measures furthermore illustrate the importance of investigating both frequency and length to infer preference. This can help to get a more realistic overall understanding of how the participating animals interact with the technology and their overall interactive behaviours and patterns.

However, even with an animal-centred approach, autonomy and consent ensured, and the information carefully interpreted, the problem of determining whether an animal “likes” a system remains, as Ritvo et al. [35] note. In our study, the sakis consistently used the system over a long period of time, thus we can assume the sound was at least not completely aversive to prevent the sakis from totally using the form. However, this does not necessarily mean that they found the sound enjoyable to listen to in the human sense of the word *liking*. One possibility is that, while the sakis preferred the *traffic* audio, they merely tolerated the other sounds to use the tunnel form itself. One indication of this could be that when the device played any other audio than the *traffic* sound the sakis’ interactions became shorter, which could be an indication of avoidance. If this was the case, it would indicate the tunnel form itself was valued by the sakis so highly that they aimed to continually use the structure despite potentially uncomfortable sounds they have to tolerate while inside. However, further work would need to be done to explore how the artefacts that provide animals with enrichment or enhancements shape the animals’ behaviours. This is a key point of researchers who design and build animal–computer systems to further untangle the devices form from the interactivity.

Another potential explanation is that the sakis simply prefer to use the system differently when it plays audio. Regardless, as there were shifts in usage patterns (interaction length and frequency) between different sounds as well as *silence*, it would be fair to say that the present audio (or lack of one) affected how the sakis interacted with the system and that they sought to trigger certain audios (traffic in particular) more than others. This does, however, indicate that, even if we can never fully understand the animals’ experience and whether this preference is akin to the human notion of “liking” a system, we can get a sense of the animals’ preferences (even if not the motivation) by using our presented method. This information then can be used to begin building up an understanding of what sakis’ audio preferences and behaviours are and how an animal’s interaction reflects their preferences and shapes their technology usage and forms. Equally, this highlights the importance that form and aesthetic plays when forming animal technology systems.

### 4.3. Limitations and Future Directions

As this is a case study focusing on one group of animals, our results have limitations in generalisation and sample size and are placed within the time and context that the research was conducted within. Moreover, we did not analyse the behaviours of different individuals separately, which could have informed our results further (e.g., it is possible that one saki could have not used the system at all). Equally, the order of the tested sounds was not randomised, so even though the order was arbitrary and mitigated against by long testing times, it is possible that there could be an ordering effect. Additionally, our system occasionally ran out of power when the keepers forgot or did not have time to change the batteries, even after the system was lowered to a more convenient position. This was the most prominent practical issue in our case of using a self-contained system in the middle of an active zoo enclosure, meaning it had to run on battery power. To create comparable results, the data were averaged over the test days for the statistical test, and otherwise normalised by the total number of hours. These problems in keeper resources and changes in the environment are challenges that are difficult to avoid when working in a live zoo environment over a period of several months. Thus, while our method demonstrates the benefit of having a system within the animals’ enclosure, future work would need to have systems that require little to no keeper involvement to maintain a stable system.

In the future, it would be beneficial to systematically investigate different audio features of sounds with sakis and to analyse the sakis’ overall individual and group behaviours more systematically, such as using ethograms. This would generate more in-depth behavioural results that would allow drawing conclusions about the enrichment benefits of such a system and different sounds. Here, the behaviours were only briefly investigated within the scope of technology interaction, with the focus mainly on the interaction data. Furthermore, even though the research coordinator and the zookeepers were involved throughout the project to ensure the system was appropriate for the sakis and their welfare was not compromised, we did not have in-depth involvement of an animal behaviour specialist during the whole process. As such, we can only state observations on the interaction frequency and length, not whether they resulted in any enrichment benefit. We also only focused systematically on the interactions within the prototype after adding the sound, so any changes to the overall interactions with the prototype (behaviours on or around it, or changes in the behaviour inside the tunnel beyond the time spent in) were not tracked.

Lastly, our method is by no means the only possible approach to study sound preferences or interaction modalities with primates. However, for us, ensuring the voluntary participation of the animals was important, as this is one of the central premises of ACI. To achieve this, it was necessary to have a system the sakis would trigger themselves, as there was no way of adding sound to their environment in such a way that they could have completely avoided it. In this sense, the sound and the device and interaction system were intimately intertwined. In future work, there could be value in using more focused approaches to investigate particular aspects of this kind of a complete system, such as isolating and experimenting only with sounds or different interaction mechanisms.

For the wider animal–computer community, our method could be generalised to be used with any animals in zoo context, and also other ACI contexts with appropriate modifications. The questionnaire can be utilised to form initial requirements specific to a particular animal species. For us, it allowed us to collect the perspectives of several keepers when they had very limited time to participate in activities beyond their daily duties such as this kind of a study. The visitor questionnaire is not necessary from the animal-centred point of view. Zoos, however, still have incentives to ensure good visitor experience and potential negative views of visitors can be relevant when it comes to technology use of the animals, as gauging the visitor attitudes can be important for the zoo before deploying new systems. Early prototyping and comparison of different aspects of the system can help guide designers when the animals’ preferences are not clear from previous experience, as well as reveal new insights even when collaborating with experts who have an in-depth understanding of the animals. In this way, our method can be used as a tool for participatory design. Forming the interaction mechanisms of the device based on observed behaviour especially can help avoid falling into typical human-centred ways of interaction, such as touch screens or buttons which may not be optimal for many animals. Using proximity detection or physical presence like we did here can be one straightforward way to initiate simple interactions, but we acknowledge that these would not be sophisticated enough for more elaborate use cases. However, regardless of the interaction mechanism used, automatic tracking of the system use and activation of the interaction can be leveraged to gain a more comprehensive understanding of the interaction behaviours and patterns, especially over a long period of time. Within the scope of this study, we focused purely on this usage data, but most benefits can likely be acquired by combining it with more systematic behavioural observation such as ethograms and further behavioural analysis.

## 5. Conclusions

In this paper, we form a method to build an interactive audio device that allows white-faced sakis to use it within their zoo environment (RQ1) and for us to explore what audio they choose to listen to (RQ2). This system was designed with the sakis by prototyping different forms from zookeeper and visitor requirements and zookeeper expertise and building an interaction method from these early interactions. We then installed the audio system within the sakis’ zoo habitat, allowing them to control their own audio with the system while automatically recording their usage. Using a baseline week without audio, we then enabled the system with various audios over several weeks (rain, zen, traffic and electronic music) and recorded the system activations triggered by the sakis. Towards our first aim, our results indicate that the sakis had preferences toward the tunnel prototype structure over the dome structure, which were revealed by testing and including the sakis already early in the process. The early inclusion, as well as automatic monitoring, allowed us to observe how the sakis ordinarily interact with the structures, which we used to direct our later design decisions. This method contributes a deeper way of understanding and highlights the benefits of including the animals to the design directly and early within the process. Furthermore, our work illustrates how we can provide the animals with autonomy and consent over their usage of a proximity triggered system when studying their responses to audio stimulus. Towards the second aim, our results also reveal how important it is to complement this with longitudinal testing to ensure core facets of the animals’ experience and interactions do not remain undiscovered. Here, we found that the sakis chose to listen to traffic noise over silence and rain, zen and music sounds. We note, however, that the sakis’ interactions (frequency and length) with the device vary with audio compared to without. The insights found in this study would benefit those developing animal-interactive audio enrichment and those working to design animal technology in a zoo environment. Importantly, the work here adds to methods, especially around early design methods, in constructing animal-centred and animal friendly methods that facilitate animals using and controlling computers. Our system, and method, is the first that the authors know of that allows primates to control their own audio system within their environment over several weeks. While these contributions are imperative towards designing interactive technologies for non-human animals, we also present guidelines for researching and designing with animals in their ordinary zoo environment questioning what it means to interact as an animal with computer systems.

## Figures and Tables

**Table 1:** Requirements identified from our questionnaire consisting of shared (common) requirements by visitor and keepers and keeper specific requirements. Ordering indicates descending priority.

Common Requirements	Keeper Requirements
CR1. Enrichment	KR1. Not easily breakable
CR2. Health	KR2. Not too frustrating
CR3. No negative effects	KR3. Not too easy
CR4. No training required	KR4. Not addictive
CR5. Can see the animal using	KR5. Needs to be monitored

**Table 2:** Codes describing interactions with the prototype and percentage of each instance over all interaction events. Codes are split by animal placement for the interaction, behaviour, social usage and other codes. Codes are shown in %.

Tunnel (N = 677)							
**Inside**	**19.9**	**Behaviours**	**10.8**	**Social**	**5.8**	**Others**	**85.4**
go through	17.7	grab	9.6	groom	1.0	go over	55.0
sit on edge	7.1	bite	3.3	shoo	1.6	on top	26.0
sit middle	0.9	look through	0.4	multiple individuals	5.8	next to	8.1
						food	1.3
**Dome (N = 54)**							
**Under**	**83.3**	**Behaviours**	**33.3**	**Social**	**0**	**Others**	**9.3**
		grab	18.5			next to	9.3
		bite	11.1				
		touch	14.8				

**Table 3:** Results of the Wilcoxon signed rank-sum test for mean interaction time per 30 min with $H_{0}$ that the median of differences is 0, the alternative hypothesis $H_{1}$ being that it is positive, i.e., A is preferred over B. Effect size *f* is given as fraction of pairs that support the alternative hypothesis. * *p* < 0.5, ** *p* < 0.01.

A/B	1. Silent	2. Rain	3. Zen	4. Traffic	5. Electronic Music
1. silent	-	W = 155.5, *p* = 0.432, f = 0.417	W = 141.0, *p* = 0.596, f = 0.333	W = 66.0, *p* = 0.991, f = 0.208	W = 140.0, *p* = 0.612, f = 0.458
2. rain	W = 143.5, *p* = 0.568, f = 0.542	-	W = 121.5, *p* = 0.788, f = 0.333	W = 61.0, *p* = 0.994, f = 0.291	W = 143.0, *p* = 0.574, f = 0.458
3. zen	W = 158.0, *p* = 0.404, f = 0.625	W = 177.5, *p* = 0.212, f = 0.625	-	W = 49.0, *p* = 0.998, f = 0.292	W = 173.0, *p* = 0.256, f = 0.583
4. traffic	W = 233.0, *p* = 0.0085 **, f = 0.750	W = 238.0, *p* = 0.0057 **, f = 0.667	W = 250.0, *p* = 0.0020 **, f = 0.667	-	W = 226.0, *p* = 0.015 *, f = 0.625
5. electronic music	W = 160.0, *p* = 0.388, f = 0.542	W = 156.0, *p* = 0.426, f = 0.500	W = 127.0, *p* = 0.744, f = 0.417	W = 74.0, *p* = 0.985, f = 0.375	-

**Table 4:** Use time, duration, and frequency of interactions the sakis had with the audio enrichment device playing different sounds. Results reported in seconds (s) or interaction numbers.

	Frequency	Use Time (s)	Duration (s)				
	No. per hour	Total Use per hour	Mean	Median	Std	Longest	Shortest
Silent	1.84	43.00	23.35	4.0	107.50	1052	1
Rain	2.37	14.79	6.24	4.0	11.40	116	1
Zen	2.61	21.14	8.11	3.0	16.07	141	1
Traffic	3.90	112.16	28.77	6.0	73.79	882	1
Electronic music	2.00	19.75	9.87	3.0	33.29	307	1

## Appendix A

Data and code as well as the requirement gathering questionnaire are available on Github: https://github.com/roosapi/music-for-monkeys.
